# Dichotomous assessments of homologous recombination deficiency are insufficient predictive biomarkers for the PARP inhibitor treatment of ovarian cancer

**DOI:** 10.1002/ctm2.70890

**Published:** 2026-11-01

**Authors:** Jesenia M. Perez, Justin Hwang, Britt K. Erickson, Stefani N. Thomas

**Affiliations:** ^1^ Department of Medicine University of Minnesota School of Medicine Minneapolis Minnesota USA; ^2^ Department of Obstetrics Gynecology and Women's Health, University of Minnesota School of Medicine Minneapolis Minnesota USA; ^3^ Department of Laboratory Medicine and Pathology University of Minnesota School of Medicine Minneapolis Minnesota USA

1

Predictive biomarkers of disease are essential for treatment stratification, prognosticating, and developing strategies to improve outcomes. Assessment of *BRCA1/2* mutation status and homologous recombination deficiency (HRD) are the current approaches used to designate maintenance treatment for epithelial ovarian cancers (EOC).^1^ The high recurrence rate and poor 5‐year survival underscores the importance of comprehensively defining one of the key characteristics of epithelial ovarian cancer: genome instability. With the development of sophisticated multi‐omics techniques, the field is well‐positioned to expand current dichotomous assessments of HRD to improve the utility of poly (adenosine diphosphate‐ribose) polymerase inhibitor (PARPi) therapies in ovarian cancer.

The primary treatment for advanced‐stage ovarian cancer is guided by disease staging from surgery, tumour histotype and primary site, *BRCA1/2* mutation status, HRD status and patient‐related factors such as functional status and comorbidities.^2^ After surgical cytoreduction and completion of primary platinum‐based chemotherapy, patients with tumours harbouring *BRCA* gene variants or HRD can also receive PARPi maintenance therapy (olaparib, niraparib) alone or in combination with the angiogenesis inhibitor, bevacizumab (Table 1).^1^ Patients whose tumours are HRD negative (also known as Homologous Recombination Proficient (HRP)) are given bevacizumab or no further treatment in the maintenance setting.^2^ Despite advancements in the use of neoadjuvant chemotherapy and individualised maintenance therapies with PARPi, disease recurrence is observed in 70% of patients within 3 years of first‐line chemotherapy. Additionally, treatment with PARPi frequently leads to the development of haematological toxicities including anaemia, thrombocytopenia, and neutropenia; these adverse events are the main contributors to interruption and discontinuation of PARPi treatment, and they impact patients’ quality of life.^3^ Furthermore, PARPi therapy may increase the risk of fatality due to myelodysplastic syndrome and acute myeloid leukaemia.^4^ The likelihood of the occurrence of these and other adverse events emphasises the importance of appropriate PARPi‐treatment stratification in maintenance therapy to reduce disease recurrence, prolong survival, and improve patient quality of life.

**TABLE 1:** Maintenance therapy for newly diagnosed advanced stage EOC based on NCCN Guidelines Version 4.2026.

Indication	Genetic feature	Maintenance therapy
**Newly diagnosed advanced stage EOC**	Germline or somatic *BRCA1/2* pathogenic/likely pathogenic variants	If Bevacizumab not used during primary chemotherapy: Olaparib, Niraparib, or Rucaparib
		If Bevacizumab was used during primary chemotherapy: Olaparib + Bevacizumab, Niraparib + Bevacizumab, Olaparib, Niraparib, or Rucaparib
	*BRCA1/2* wild‐type or unknown and HR Deficient	If Bevacizumab not used during primary chemotherapy: Olaparib, Niraparib, or Rucaparib
		If Bevacizumab was used during primary chemotherapy: Olaparib + Bevacizumab, Niraparib + Bevacizumab, or Bevacizumab, or Olaparib
	*BRCA1/2* wild‐type or unknown and HR Proficient or Unknown HR status	If Bevacizumab not used during primary chemotherapy: Observe or therapy for persistent disease or recurrence
		If Bevacizumab was used during primary chemotherapy: Bevacizumab

PARPi function by inhibiting single‐strand DNA break repair by PARP1 and PARP2 enzymes, leading to the accumulation of mutations and the formation of double‐strand DNA breaks.^5^ In tumour cells with non‐functional *BRCA1/2* or HRD, double‐strand DNA breaks are repaired through the error‐prone non‐homologous end joining (NHEJ) pathway, leading to chromosomal instability, cell cycle arrest, and ultimately cell death. This synthetic‐lethal interplay between PARP inhibition, *BRCA1/2* alterations, and HRD phenotypes (known as *BRCAness*) has proven efficacious in clinic through improvements in progression‐free survival (PFS) and overall survival (OS).^6^ For patients with advanced‐stage ovarian cancer, PARPi treatment with olaparib, niraparib, or rucaparib was assessed as maintenance therapy after response to platinum‐based chemotherapy in multiple phase 3 randomised clinical trials. Results from the SOLO1 trial showed that olaparib improved long‐term (7 years) OS in patients with *BRCA1/2* variants as compared with placebo.^7^ Similarly, the PAOLA‐1 trial demonstrated that olaparib plus bevacizumab improved median OS versus bevacizumab treatment alone in patients that are HRD positive.^8^ The PRIMA trial observed improvements to median PFS with niraparib treatment for individuals who were HRD/*BRCA*‐mut versus placebo (30.1 vs. 11.5 months, HR 0.43), and HRD/*BRCA*‐wt versus placebo (19.4 vs. 10.4 months, HR 0.67).^9^ Importantly, there was a slight improvement in the HRP group treated with niraparib as compared to placebo (8.4 months vs. 5.4 months, HR 0.67), indicating that in these patients there was some level of HR deficiency not detectable by the current clinical assay. This indicates that the HRD biomarker needs to be refined to include patients who are currently testing negative but would derive benefit from PARPi treatment. A significant limitation to the findings of these studies, however, is that each trial was comprised of heterogeneous populations of patients utilising distinct HRD tests, which makes it difficult to appropriately assess the efficacy of current treatment stratifications.

Germline *BRCA1/2* mutations occur in 15% of high‐grade serous ovarian cancers (HGSOC) while 6% of cases exhibit somatic mutations. Both somatic and germline mutations in other genes involved in HR including, but not limited to, *TR, ATM, RAD51/54, CHK1/2, PALB2*, and *PTEN* occur in 20% of HGSOC cases.^10^ Current FDA‐approved HRD tests commonly used in this setting, myChoice CDx and FoundationOne CDx, broadly report HRD through a combined genome instability score representing loss of heterozygosity (LOH), telomeric allelic imbalances (TAI), and large‐scale transitions (LST) using whole genome sequencing (WGS) and single nucleotide polymorphism (SNP) assays (Table **2**). Through this definition of genome instability, HRD positivity is found in 50% of HGSOC cases. HRD‐associated mutational profiles focus on the presence of copy number alterations, single‐nucleotide variants, and indels of HRR genes. Collectively, these mutations are referred to as genomic scarring. Commercial HRD assays such as myChoice CDx analyse SNPs of *BRCA1/2* using an algorithm that sums features of genomic instability, while FoundationOne CDx utilises a gene panel composed of 324 genes (including *BRCA1/2*) and solely calculates LOH.^6^ Consequently, these two FDA‐approved HRD assays demonstrate differences in reported HRD thresholds (myChoice CDx: ≥42, FoundationOne CDx: ≥16%) and cannot be treated equivalently.^11^ In comparing the two FDA‐approved assays, a study by Timms *et al.* found that 19‐61% of patients considered HRD positive through the myChoice CDx would have been considered HRD negative when considering LOH alone.^12^ A robust HRD signature encompassing genetic alterations to HRR genes, epigenetic alterations, and functional components is needed to not only better stratify patients for PARPi treatments but also measure variations in HRD phenotypes that can be influenced by current treatment strategies.^13^

**TABLE 2:** FDA‐approved HRD tests for clinical use.

Test	Company	Gene panel	Biospecimen type	Method	Positive HRD result
MyChoice CDx	Myriad Genetics	*BRCA1* and *BRCA2*	FFPE tumour tissue	Detects SNVs, large rearrangements, germline, and tumour pathogenic variants in *BRCA1/2*. Genome instability scores (GIS) calculated by loss of heterozygosity (LOH), telomeric allelic imbalance (TAI), and large‐scale transitions (LST).	*BRCA1/2* mutation and/or GIS ≥ 42
FoundationOne CDx	Foundation Medicine	324 genes, including *BRCA1* and *BRCA2*	FFPE tumour tissue	Detects substitutions, indels, and copy number alterations. Analyses genomic rearrangements, microsatellite instability, and tumour mutational burden. Calculates genomic loss of heterozygosity (gLOH).	*BRCA1/2* mutation and/or gLOH ≥ 16%

Clinical assessment of HRD through integrated multi‐omics analysis of patient molecular profiles is required to refine the use of PARPi for patients with HGSOC. Genomics‐based HRD classifier tools have previously been used to improve clinical designations of HRD. Classifier of Homologous Recombination Deficiency (CHORD) is a WGS‐based tool that used a random‐forest model from 3584 patients to determine HRD status across different cancer types.^14^ While this model can distinguish between *BRCA1* and *BRCA2*‐associated HRD, it is limited to characterising genomic scarring and does not consider functional features of deficient DNA repair. Attempts at characterising HRD via transcriptomic signatures from public data portals including The Cancer Genome Atlas (TCGA) have shown variable correlations between genomic and functional indicators of HRD in a cancer type‐dependent manner. For example, Lee *et al.* established a machine‐learning based model trained on transcriptomic data from TCGA and showed that Pearson correlation coefficients of HRD scores predicted by their model versus scarHRD (measuring genomic instability) were 0.858 for pan‐cancers and 0.768 for ovarian cancer.^15^ However, models that attempt to characterise HRD from TCGA data can be confounded by distinct therapies, treatment cycles, and patient ages since TCGA contains data from patients receiving routine clinical care rather than enrolled in controlled clinical trials.^16^

Other efforts have integrated clinical and molecular data to predict PARPi efficacy. A retrospective study by Marchetti *et al.* assessed *BRCA*/HRD status, chemotherapy response scores (CRS), and CA‐125 elimination rate constant K (KELIM) scores to identify predictors of PARPi efficacy and improve patient stratification. Although the study was hypothesis‐generating, CRS was found to be a prognostic factor of PARPi efficacy, independent of *BRCA*/HRD status in PARPi‐treated patients, correlating with PFS. Integration of these clinical and molecular features led to the development of a recurrence risk‐stratification model of high, high‐intermediate, intermediate, and low risk patients who may benefit from PARPi treatment.^17^ Still, these analyses require follow up with larger sample sizes and model validation.

Overall, these studies indicate an urgency to develop a robust, clinically relevant model that integrates multimodal features of HRD specific to ovarian cancer. With the current therapy paradigm for advanced‐stage ovarian cancer, many patients receive PARPi as a part of their treatment plan. A consistent and accurate HRD definition that takes into consideration molecular features of ovarian tumours (including genetic, epigenetic, and functional components) and integrates clinical features driving disease progression could reliably predict response to PARPi. Furthermore, a diagnostic test assessing these molecular features using a non‐invasively collected sample, such as a liquid biopsy, would be advantageous. Developing a more efficacious PARPi risk‐stratification model is essential for patients who would not benefit from long‐term maintenance therapy. Improved PARPi stratification would also reduce disease recurrence—a major clinical problem affecting 70% of individuals diagnosed with advanced‐stage ovarian cancer.
